# Risk-based Vaccines and the Need for Risk-based Subnational Vaccination Strategies for Introduction

**DOI:** 10.1093/cid/ciaa483

**Published:** 2020-07-09

**Authors:** Farzana B Muhib, Clint J Pecenka, Anthony A Marfin

**Affiliations:** Center for Vaccine Innovation and Access, PATH, Seattle, Washington, USA

**Keywords:** subnational, vaccine, strategy, risk-based

## Abstract

**Background:**

Most vaccines in the Expanded Program on Immunization are universal childhood vaccines (eg, measles and rotavirus vaccines). Other vaccines such as typhoid conjugate (TCV) and Japanese encephalitis vaccines are risk based and only used in countries where populations are at risk of these diseases. However, strategies to introduce risk-based vaccines are becoming complex due to increasing intracountry variability in disease incidence. There is a need to assess whether subnational vaccine strategies are appropriate.

**Criteria, challenges, and benefits:**

Subnational strategies consider intracountry heterogeneous risk and prioritize vaccination only in those areas that are at risk; there is no intent to introduce the vaccine nationally. The following variables should be considered to determine appropriateness of subnational strategies: disease burden, outbreak potential, treatment availability and costs, cost-effectiveness, and availability of other preventive interventions. We propose criteria for each variable and use a hypothetical country considering TCV introduction to show how criteria are applied to determine if a subnational strategy is appropriate. Challenges include granularity of disease-burden data, political challenges of vaccinating only a portion of a population, and potentially higher costs of introduction. Benefits include targeted reduction of disease burden, increased equity for marginalized populations, and progress on development goals.

**Conclusions:**

In the absence of perfect information at the national level, adopting a subnational vaccine strategy can provide country decision makers with an alternative to national vaccine introduction. Given the changing nature of communicable disease burden, subnational vaccination may be a tool to effectively avert mortality and morbidity while maximizing the use of available health and financial resources.

Global communicable disease burden is changing. To reduce the burden of communicable diseases, vaccination strategies will need to adapt to accommodate these changes. Established in 1974, the Expanded Program on Immunization (EPI) focused on immunization programs that ensured all children have equal access to vaccines against the most common and fatal of childhood diseases—diphtheria, tetanus, pertussis, measles, polio, and tuberculosis [1]. The key feature of these vaccines is that they are universally recommended for all children in all countries. For example, initial measles vaccination activities sought to introduce routine measles vaccination to every country in the world through the EPI and the UNICEF (United Nations Children’s Fund)-led initiative for Universal Childhood Immunization in 1990, where the recommendation was for 1 dose of measles vaccine to be administered to at least 80% of children aged 9 months or older [2]. Subsequently, several new vaccines—including hepatitis B, *Haemophilus influenzae* type b, pneumococcus, rotavirus, and human papillomavirus vaccines—were added to EPI schedules. Like measles, the goal has generally been to introduce these new vaccines to all children in all countries. For these pathogens, in an unvaccinated population, a child’s risk of infection is approximately the same across an entire country, as is the benefit of vaccination. As a result, the vaccination strategy has been relatively uncomplicated because the goal is to vaccinate all children.

In contrast, there are infections that were once globally distributed but now only occur in specific settings or in specific populations and, as a result, are often unevenly distributed within a country. Examples of such infections are typhoid and cholera. In addition, there are infections that only occur in certain regions of the world. Examples of geographically restricted, vaccine-preventable diseases include yellow fever, meningococcal disease, Japanese encephalitis (JE), and tick-borne encephalitis. A key feature of all these diseases is that the risk of disease is not uniform across all children. Unequal risk distribution of disease and a highly variable benefit of vaccination exist between countries, as well as within a country. In such cases, vaccination may not be optimally recommended for all children. These are defined as “risk-based vaccines,”which require risk-based vaccine strategies for introduction. In addition to typhoid, JE, and the other risk-based vaccines listed above, many new vaccines under development are risk-based vaccines (eg, *Plasmodium falciparum* malaria, *Shigella*). In contrast to the relatively uncomplicated vaccination strategies used to introduce measles and pneumococcal vaccines, strategies to introduce risk-based vaccines may be more difficult to develop due to increasing intracountry variability in disease incidence and the related need for more granular disease-surveillance data to target vulnerable populations.

In this article, we propose and discuss criteria that can be used to determine if a specific type of risk-based vaccination strategy—ie, a subnational vaccine strategy for risk-based vaccines—should be considered by a country, as well as the challenges and benefits of adopting such a strategy.

## HETEROGENOUS DISTRIBUTION OF DISEASE: A NEW CHALLENGE

Despite the heterogenous distribution of these diseases, with only 1 or 2 exceptions [3, 4], current World Health Organization (WHO)–recommended risk-based vaccines have still been introduced nationally, averting many of the hard decisions that must be made when vaccines are introduced in some populations and not others. As vaccine cost and variability in disease burden increase, this is unlikely to be possible for all future introductions. Similarly, as vaccine hesitancy increases, parents and healthcare providers will be more demanding of evidence that each child will benefit from a vaccination in order to offset any perceived risk, making risk-based vaccination more attractive in this context.

One challenge of risk-based vaccination is defining the heterogenous distribution of disease within a country. Traditionally, this intracountry heterogeneity has been an acknowledged feature for mosquito-borne diseases like malaria [5], where the presence of virus- or parasite-infected vectors is dependent on rainfall patterns, altitude, breeding habitat, and immune status of amplifying hosts.

However, another type of intracountry disease heterogeneity is emerging, which may have a large impact on the distribution of disease—that is, uneven distribution of economic and infrastructure resources resulting in increased human migration, changing settlement patterns, poor access to clean water and sanitation, and economic disparity. This heterogeneity is exacerbated by increasing antibiotic resistance and a lack of access to affordable healthcare, clean water and sanitation services, and other preventative interventions [6, 7].

Intracountry heterogeneity for vaccine-preventable diseases may make adoption of a subnational vaccination strategy more appropriate. We define risk-based vaccination strategies as those that consider whether a population is at risk of a disease at the regional, national, and subnational levels. Consequently, subnational vaccination strategies are a subset of risk-based strategies. Subnational strategies consider intracountry heterogeneity risk of diseases and prioritize vaccination only in those areas at risk, instead of considering regional or national introduction of vaccines; it restricts vaccination based on specified criteria.

Many countries implement a phased vaccine introduction due to large populations or geographic challenges. For example, in 2019, typhoid conjugate vaccine (TCV) was introduced in Sindh province, Pakistan [8], which was started in 2019 with the intent to introduce the vaccine to the entire country over a 3-year period; this is still considered a national introduction strategy. However, in a subnational introduction strategy, there is no intent to introduce the vaccine nationally. An example of a subnational introduction is India’s introduction of JE vaccine [3]. Since 2006, India has expanded its JE vaccination program based on passive JE surveillance to detect new cases in children. When a JE case is identified in a district, that district is then classified as JE endemic and eligible for a one-time, catch-up campaign followed by introduction of JE vaccine into the routine immunization program.

The introduction of risk-based vaccines that result in subnational introduction requires highly granular disease-surveillance data to identify areas or populations at risk. In addition, the cost of illness, the cost of vaccine delivery, access to other disease interventions, and access to disease treatment at the subnational level should be evaluated when considering a subnational vaccination strategy. Also, factors such as the cost of reaching vulnerable or hard-to-reach populations most at risk of a disease need to be balanced with the potential to increase equity across a population by ensuring access to public health interventions.

To illustrate how a subnational approach could be used in decision making, we describe a hypothetical country that is considering TCV introduction. To be clear, we are not advocating for subnational TCV introduction. Instead, we suggest that countries with highly heterogenous disease-burden distribution may consider the criteria listed in this article when considering a subnational vaccination strategy. To date, we are not aware of any country that plans to use TCVs subnationally as a part of their routine immunization program.

## EXAMPLE OF A HYPOTHETICAL COUNTRY CONSIDERING TCV INTRODUCTION

In this example, we consider possible TCV introduction in a lower-middle-income, subtropical country with coastal and mountainous regions and substantial tracts of arable land prone to periodic flooding. The economy has been growing steadily, with most economic growth occurring in urban centers. A lack of jobs in rural areas leads to urban migration of adults who send money back to their families, as well as migration of whole families. Because of the rapid urban growth due to rural-to-urban migration, infrastructure investment is minimal and access to clean water and sanitation is limited in the growing urban slums. Because antibiotics are readily available, self-medication is common before seeking care and, as a result, antibiotic resistance is common for many bacterial pathogens in the country.

Active disease surveillance finds much evidence of typhoid in urban centers, including multidrug- and drug-resistant typhoid strains. For the sake of this example, typhoid is infrequently reported from rural areas, but it is unclear if this is due to poor typhoid surveillance or whether typhoid incidence is truly low. The country is also facing Gavi graduation and will be expected to share a greater portion of the cost of new vaccine introduction. Therefore, policymakers would like to ensure that children most at risk of typhoid receive TCV without disrupting the procurement and delivery of other childhood vaccines while ensuring that the country’s financial resources are used optimally.

## CRITERIA FOR SUBNATIONAL VACCINE INTRODUCTION

When countries consider introducing a vaccine subnationally, disease burden, outbreak potential, treatment costs and availability, cost-effectiveness, and the availability of other preventive interventions should be examined. In Table 1, we have listed the variables and criteria that could be considered when deciding to introduce a vaccine subnationally, as well as indicators present in our example that fit these criteria.

**Table 1.** Criteria and Indicators for Subnational Vaccine Introduction Strategy Determination

Variable	Criteria Subnational Vaccine Introduction Strategy	Indicators for Subnational Vaccine Strategy: Hypothetical Country Example
Disease burden	• Disease is only found in specific areas/populations, or in specific environmental contexts within countries. • Resistance to drugs to treat disease exists only in certain areas within a country. • Internal migration is limited among at risk populations or confined to administrative areas	• Disease present in urban areas, majority of disease in urban slum areas. • Disease is only detected in flood-prone rural areas. In the rural mountainous regions, or non–flood-prone arable regions there is no typhoid detected. • Antibiotic resistance detected in urban areas. No antibiotic resistance was detected in rural flood-prone areas. • Internal migration of both adults and whole families occurs but the proportion of each is not known
Potential for outbreaks	• Potential for outbreaks is concentrated in areas that have a specific set of environmental or exposure/risk characteristics.	• There are no current outbreaks of disease. However, during the rainy season, areas that are prone to flooding have experienced localized outbreaks.
Treatment availability and costs	• Availability of treatment is scarce in certain geographic areas and/or the associated costs are high for those most at risk of disease.	• Access to healthcare is low in urban slum populations, with public service seen as low quality, leading parents to seek out more expensive private care. • Antibiotics are losing their effectiveness; last-line antibiotics are being used, which are very expensive.
Cost-effectiveness	• The vaccine is only cost-effective in certain high- to medium-disease-burden areas.	• Given the distribution of disease burden, vaccine cost-effectiveness is highest in urban areas.
Other interventions	• Limited or no interventions exist to prevent the disease in the country. • The other interventions are decreasing in effectiveness to prevent/treat disease. • Other interventions are very expensive and therefore only available to certain populations. • Interventions may take considerable time to implement across the country.	• Access to clean water and sanitation services is limited, with marginalized populations in urban areas with less access than other populations in the country. • Antibiotics are losing their effectiveness.

In the case of typhoid, the presence of significantly elevated typhoid risk will ultimately determine if TCV is needed and, if that elevated risk is highly localized, whether a subnational vaccination strategy is suitable to prevent disease. To determine if typhoid burden heterogeneity exists within a country, typhoid incidence over the prior years as well as modeled estimates should be evaluated. Common data sources are surveillance systems, both published and unpublished data from research studies, and gray literature found in the country. Another option is the use of disease-specific rapid assessment tools/frameworks developed by WHO or the US Centers for Disease Control and Prevention to provide countries with guidance on how to rapidly estimate disease burden in the absence of longstanding surveillance data. A risk-assessment tool is currently under development for typhoid but already exists for other diseases [9, 10]. Finally, modeled estimates of disease burden are becoming available at the subnational level for many diseases [11] and could be a supplementary source of information.

In addition to typhoid burden heterogeneity, it is important to consider drug resistance and internal population migration patterns. Drug-resistance patterns, too, can be highly localized and may put certain groups more at risk within a country. In areas where there is high-level resistance, treatment options will be limited and prevention becomes much more important [12, 13]. Internal migration, especially of children, can warrant vaccination in areas that do not yet have disease risk. Both factors impact where a subnational strategy is pursued, with drug resistance helping to prioritize vaccination in specific populations and internal migration focusing on either the location of a subnational vaccination strategy or expanding the area in which it is implemented. In 2018, the use of TCV in Hyderabad, Pakistan, before the decision was made to introduce TCV nationally was due to the presence of extremely drug-resistant typhoid (XDR) in an urban setting and is an example of prioritizing vaccination of children at risk of XDR typhoid [14].

In our hypothetical country, there are multiple data sources that show greatly elevated typhoid incidence in urban areas but only periodic typhoid outbreaks in the rural areas prone to flooding. High levels of antibiotic-resistant typhoid are present in urban areas but not the flood-prone rural areas. Internal migration of both adults and whole families to urban areas will need to be assessed to understand the impact of a subnational strategy.

While the potential for typhoid outbreaks may be considered as part of typhoid disease burden, the potential for outbreaks warrants its inclusion as a separate variable in Table 1. Typhoid and other enteric disease outbreaks occur for a variety of reasons, including flooding, drought, poor water and sanitation infrastructure, and poor water, sanitation, and hygiene practices [15–18]. In our example country, there are typhoid outbreaks in rural areas following floods that occur on a regular basis.

The next variable in Table 1, treatment availability and costs, can be dependent on location and/or socioeconomic status [19, 20]. These 2 factors usually impact marginalized and/or rural populations to a greater extent, so that they do not receive adequate care. Prevention via vaccination may have a greater impact on severe disease and mortality in these populations. In our example, the population most at risk has limited access to public services, forcing them to seek out private healthcare and self-medication. In addition, antibiotic resistance has made treating typhoid more expensive.

Cost-effectiveness is another factor that may be considered in pursuing a subnational vaccine strategy. Disease burden and price of the vaccine are 2 main drivers of cost-effectiveness. One example of how cost-effectiveness may be used to inform subnational vaccine strategy is an evaluation of the RTS,S malaria vaccine [21]. The authors produced cost-effectiveness estimates for a range of parasite prevalence settings that are correlated to underlying disease burden. They determined the vaccine was cost-effective if used in parasite prevalence settings of greater than 10%. This finding may be used by country decision makers to prioritize children living in parasite prevalence settings of greater than 10% to receive the vaccine. In the context of enteric diseases, a recent paper looking at the subnational vaccine impact and cost-effectiveness of hypothetical enterotoxigenic *Escherichia coli* and *Shigella* vaccines starts to highlight how subnational cost-effectiveness could be used in decision making [22]. In our example country, the introduction of TCV would be most cost-effective in urban areas.

When considering vaccination, it is important to evaluate other available interventions, their effectiveness, and how long they may take to implement and have impact (Table 1). For pathogens where more than 1 intervention effectively prevents disease, a vaccine may not always be the first consideration. As with other factors, there is variability in the effectiveness of other interventions at the subnational level. As a result, subnational vaccination may be considered in areas where other interventions are considered ineffective but not in areas where such interventions are effective. In our example, investment in clean water and sanitation systems in urban areas would be a good way to prevent typhoid; however, it could take years to have an impact on the typhoid burden. Due to growing incidence and antibiotic resistance, subnational vaccination could be a good short-term solution, while a parallel investment is made in infrastructure.

## IS A SUBNATIONAL VACCINE STRATEGY APPROPRIATE FOR OUR HYPOTHETICAL COUNTRY?

Based on indicators for our example country in Table 1, TCV should be introduced in urban areas with a great emphasis on urban slums and strong consideration of introducing vaccines in the areas prone to flooding and periodic typhoid outbreaks, especially considering internal migration patterns.

## CHALLENGES OF SUBNATIONAL VACCINE INTRODUCTION

The main challenge to subnational introduction faced by decision makers will be determining the regions in which a vaccine should be introduced. While there are other challenges associated with this choice, the granularity of the surveillance and disease-burden data is a fundamental factor in this decision.

In our example, district-level typhoid incidence data are needed to inform subnational vaccination strategy suitability. Although there are multiple sources of data that can be used to determine disease-burden heterogeneity, there are no perfect data available in any country and it is important to assess the quality of the data/information upon which a decision will be based. As new data become available, a country can expand immunization to include newly identified disease endemic areas. This is a strategy that was implemented by both India and Nepal as they introduced JE vaccine. In the case of Nepal, JE vaccine was first introduced in 2006 in 6 high-disease-burden districts via a campaign [4]. The surveillance system was strengthened and led to vaccine being used routinely in 2009 in 22 districts, with 9 more districts added in 2011, and the remaining 44 districts introducing JE vaccine routinely. Nepal did not wait for data to become available from the entire country before introducing JE vaccine, and likely prevented substantial morbidity and mortality.

Political challenges are hard to predict but may be encountered. Subnational vaccination may pose political challenges from groups receiving and not receiving the vaccine. For diseases such as malaria or JE, the general population often knows if the area they live in is endemic for the disease. However, for diseases such as typhoid, the specific risk of typhoid may not be generally known. When the general population knows their risk of a disease, not introducing a vaccine creates political problems because populations not receiving a vaccine may feel marginalized. The Malaria Vaccine Implementation Program evaluation will offer some insights into this specific issue in the coming years (Dr Jessica Price, personal communication, 2019). Where the general population does not know their risk, vaccine uptake may be low, because the populations may distrust the offer of vaccination, potentially attributing sinister motives for them to be vaccinated but not the persons in adjacent districts or provinces. Distrust of vaccination may be exacerbated if disease-burden heterogeneity corresponds to ethnic or religious differences. Strong messaging around disease risk and the benefit of vaccination will be needed if a subnational vaccine strategy is to be successful. Another political challenge is the concentration of disease in marginalized or poorer segments of society. Country ministries of health and finance would have to recognize the disproportionate risk faced by this population and be prepared to use sparse funding and human resources to focus introduction of vaccines that would only serve this population and not the entire country. This may be a difficult political argument; however, as discussed below in the benefits section, focusing resources in these populations may increase equity and have donor support.

Another potential challenge is higher costs of delivery to reach target populations. Increased costs will be dependent on geographical barriers, socioeconomic barriers, or the need for additional advocacy, communication, and social mobilization. Often, those most at risk of certain diseases may be marginalized or hard to reach due to geographical or socioeconomic barriers. To reach these populations or areas, there may be a need to spend more funds; however, due to the increased impact of vaccinating these populations, vaccination may still be highly cost-effective and may serve to increase equity as discussed below.

## BENEFITS OF SUBNATIONAL VACCINE INTRODUCTION

The greatest benefit of adopting a subnational vaccine strategy is to reduce disease burden in those populations or areas at highest risk. Assuming high-quality delivery in all settings, vaccine effectiveness and impact is always greatest in high-disease-burden areas and targeting vaccine introduction to those at greatest risk will substantially decrease disease burden even when evaluated at the national level.

Another benefit is the opportunity to increase equity between populations/areas within countries. Those at highest risk of disease may be marginalized populations. While EPI systems have more equitable coverage than other intervention services, there is evidence that vaccination rates are lower in marginalized or hard-to-reach populations [23]. Targeted vaccination of these populations provides additional resources that can improve service delivery of multiple vaccines. In addition, there could be increased economic equity, as vaccination can decrease out-of-pocket treatment costs for marginalized populations. Inpatient costs for typhoid were estimated in several studies and ranged from $159 to $636, while outpatient costs ranged from $17 to $74 (US dollars) [24]. This represents a significant amount of income for most populations in lower-middle-income or low-income countries. Cost of treatment can lead to food insecurity, a loss of savings, as well as loss of income caring for a sick family member. Preventing that cost with vaccination may result in these populations being on more equitable economic footing with those populations who are not at risk of disease. While the cost of delivering vaccine may be higher to marginalized populations, this needs to be balanced with potential to increase equity, which is explored further below.

Targeted vaccination may also increase vaccine impact and cost-effectiveness. Vaccine introduction in populations at medium to high risk should prevent most of the morbidity and mortality in a country. Cost-effectiveness of the vaccine is also improved because most of the disease is prevented by only vaccinating those at high or medium risk. While cost of delivery may increase due to targeting harder-to-reach or marginalized populations, this minimal increase in cost could still result in a highly cost-effective vaccine because of the higher vaccine impact in these populations.

A subnational vaccination strategy may also help a nation to meet its development goals. Countries have made great strides in reducing communicable disease mortality and morbidity. Now, much of the disease burden is concentrated in marginalized populations or in areas with inequitable access to resources. If interventions are prioritized for introduction in marginalized populations, they could result in both prevention of disease in these populations and, as mentioned above, the allocation of additional resources to ensure these populations are getting equal access to vaccinations.

## LIMITATIONS

The criteria in Table 1 come from an analysis of the choices faced by countries when considering subnational introduction of vaccines for vector-borne diseases that the authors have worked on in the past (JE and *P. falciparum* malaria). As new information on heterogenous disease burden for non–vector-borne diseases at the subnational level becomes available, these same criteria could be used to determine if alternative vaccine strategies should be considered. However, these criteria have not yet been applied systematically in a country setting; this paper is intended to be a first step to gather and summarize criteria so that they can be considered when evaluating the appropriateness of subnational vaccine introductions strategies.

These criteria are not an exhaustive list and may not apply to every situation that a country faces. A consideration not discussed in this paper is lifelong risk of disease; understanding the potential for adult disease over the longer term can be an important factor to evaluate when adopting a subnational vaccine strategy. Lifelong risk of disease may prompt a country to consider national introduction or consider developing platforms to address life-course vaccination [25].

Another factor not discussed extensively is the importance of ongoing surveillance in areas that do not introduce vaccine. Factors including climate change, degrading infrastructure, and introduction of pathogens into areas not previously endemic may lead to areas becoming newly endemic. Ongoing surveillance will allow decision makers to evaluate if vaccines should be implemented in new areas based on disease-burden data, as well as provide information on the other criteria included in this paper.

It is not our intent to suggest that all these criteria be present for a country to adopt a subnational strategy. For example, a country may be nationally endemic for a disease but, due to the availability of other interventions or treatment, certain areas within the country experience considerably fewer to no infections. A country may decide to only introduce a vaccine in areas that do not have access to alternative interventions or treatment in order to prevent disease in these populations and maximize impact of the vaccine. Ultimately, the weight given to each criterion in order to determine the use of a subnational vaccination strategy is up to national policymakers and depends heavily on country context and resources.

## CONCLUSIONS

In this paper we propose criteria that could be considered when deciding to adopt a subnational vaccination strategy, as well potential benefits and challenges associated with this approach. As an example, we used a hypothetical country with significant typhoid burden to show how these criteria could support a subnational TCV vaccination strategy. Typhoid conjugate is just one example of a vaccine where subnational vaccination may be a preferred strategy, while still maximizing impact in the most affected populations and reducing the overall typhoid burden within a country.

In the absence of perfect information at the national level, the option of adopting a subnational vaccine strategy can provide country decision makers with an alternative to national vaccine introduction. Adopting a subnational vaccine strategy allows decision makers to address the known public health problem immediately and use this experience and future evidence to support introduction in other areas. Given the changing nature of global communicable disease burden, subnational vaccination may be a tool to effectively avert mortality and morbidity while maximizing the use of available health and financial resources.

## Notes

***Acknowledgments.*** The authors thank Laura Meyer for her assistance in preparing the manuscript for submission. We thank Duncan Steele, Aziza Mwisongo, and Lou Bourgeois for their feedback and support. We also thank the Bill & Melinda Gates Foundation.

***Financial support.*** This work was supported by the Bill & Melinda Gates Foundation (grant number OPP1180199).

***Sup*** ***plement sponsorship.*** This supplement is funded with support from the Coalition against Typhoid Secretariat, housed at the Sabin Vaccine Institute in Washington, DC and made possible by a grant from the Bill & Melinda Gates Foundation.

***Potential conflicts of interest.*** The authors: No reported conflicts of interest. All authors have submitted the ICMJE Form for Disclosure of Potential Conflicts of Interest.
